# Breast cancer surgery after the COVID-19 pandemic

**DOI:** 10.2217/fon-2020-0619

**Published:** 2020-08-20

**Authors:** Fiona Tsang-Wright, Marios-Konstantinos Tasoulis, Nicola Roche, Fiona MacNeill

**Affiliations:** ^1^Bucks Breast Unit, Buckinghamshire Healthcare NHS Trust, Queen Alexandra Road, High Wycombe, HP11 2TT, UK; ^2^Breast Surgery Unit, The Royal Marsden NHS Foundation Trust, Fulham Road, SW3 6JJ, London, UK

**Keywords:** breast cancer, breast surgery, COVID-19, recovery phase

The emergence of a novel coronavirus (SARS-CoV-2)-associated outbreak of respiratory disease (coronavirus disease 19 [COVID-19]) has developed into a global public health emergency and has been declared as a pandemic by the WHO [1]. As the number of affected individuals continues to increase with more than 15 million confirmed cases, healthcare systems worldwide have been placed under unprecedented pressures. This has had a significant impact not only on COVID-19 care but also on other acute but highly treatable conditions, including cancer. Data recently released by the UK Office for National Statistics shows a significant increase in the number of deaths compared with the previous 5-year average [2], and this is a direct consequence of COVID-19 and also the indirect impact of healthcare systems’ responses to managing the virus, including a reduction in cancer referral rates, a reduction in emergency attendances and the cancellation of elective surgery [3,4].

Within this challenging and unstable environment, the entire landscape of cancer management is rapidly evolving [5], and there are concerns that cancer care has been deprioritized or delayed [6] with implications for breast cancer, the most common female malignancy worldwide, which accounts for a significant proportion of the oncology workload. The changes center around the redeployment of the workforce, reallocation of resources and infrastructure to the critically unwell with impact on capacity, and also the reduction of the transmission risk of COVID-19 to protect manpower and reduce the R rate using a ‘reduction in footfall’ principle. Healthcare services have rapidly adapted by adopting remote or telephone consultations; deferring routine episodes such as screening survivorship patients and those with a high risk of developing breast cancer, those who have low-risk lesions, or those with noninvasive breast cancer; and halting longer exposure and resource-heavy procedures such as reconstructions and oncoplastic remodeling [7,8].

To provide a framework for breast cancer specialists, professional bodies issued guidelines on risk stratification of essential breast cancer care based on tumor stage and biology and focused on identifying patients for whom surgery was time-critical or those for whom surgical intervention could be reasonably deferred for a period of time. In early-stage breast cancer, deferring surgical management for up to 60 days does not adversely affect oncological outcomes regardless of tumor biology [9]. However, delaying surgery for 90 days in patients who are not receiving active cancer treatment is associated with a 3–4% decline in overall survival [10]. Reducing the wait to conventional definitive cancer treatment is therefore an important factor to consider after the pandemic and ideally should be scheduled within 60 days. Following current recommendations, patients for whom alternative treatment options such as neoadjuvant chemotherapy or primary endocrine treatment were not suitable would be prioritized to urgent surgery.

Working in the COVID-19 pandemic has also been likened to war due to similarities of long hours in challenging, high-pressure environments, concerns about the lack of personal protective equipment provision, and working above expertise and comfort levels in unfamiliar surroundings for a workforce already compromised by large vacancies, high turnover rates, high attrition rates, heavy workloads and increasing levels of stress. These factors compound the risk of moral injury, such as the inability to provide high-quality care and healing in the context of healthcare [11]. Healthcare workers as a result have higher rates of post-traumatic stress disorder, depression, anxiety disorders, substance misuse and suicide. The additional requirement of ‘caring’ for the healthcare workforce, on and off the frontline, with rest, time to recuperate and psychological support should therefore be the principal consideration in future planning. Individualized personalized support has been shown to be effective especially for those who have been separated from their family to protect vulnerable or shielded members; those who have been unable to access their normal support networks due to community self-isolation; those in whom relationships have been negatively impacted by a death in family, friends or co-workers; or other secondary crises. Initiatives including a ‘return to normal work’ interview, phased returns, monitoring for mental health issues with online self-check tools, offering access to professional care and structured forums such as Schwarz rounds have been shown to be effective in protecting the mental health of staff [12]. Without bold action, the Institute for Public Policy Research reports that 20% of health professionals believe that COVID-19 has made them more likely to leave the profession [13].

As the number of COVID-19 cases starts to plateau across the world, the next step is the recovery phase (i.e., restarting and increasing non-COVID-19 activity). Resuming activity to pre-pandemic levels will not be straightforward due to the continued prevalence of COVID-19 within the population and the need to adapt the ‘traditional’ ways of working to ensure the ongoing safety of patients and staff, with measures in place until a vaccination program is fully scaled up and even indefinitely. The surge of patients requiring diagnosis and treatment will be a significant factor to consider when planning the recovery phase to be able to provide optimal treatment without significant delays. This includes the backlog of patients for whom management was modified or deferred, and it is key that this group is not ‘lost’ in the rapid changes of the pandemic. The multidisciplinary coordinators and hospital trackers are integral in ensuring that these patients and their management impacted by COVID-19 are recorded and appropriately followed, with definite re-evaluation dates. The many changes to management plans that were made in a short time frame, such as stopping neoadjuvant chemotherapy, halting adjuvant radiotherapy or commencing hormonal treatment as an interim measure until surgery, have significant implications on further treatment, so keeping an accurate record is paramount. A contemporaneous record will ensure that this backlog can be actively managed once the pandemic settles, and risk stratification of the disease, comorbidities and availability of staff and facilities will determine the way through the backlog. It is important to appreciate that the decisions made within the thick of the pandemic can be re-revisited at any point and treatments that were assessed to be initially of higher risk can be re-evaluated with patients reassessed and management changed.

A second group, those with a delayed breast cancer diagnosis, will be of particular importance for forecasting future service demand. A delayed cancer diagnosis could be explained by not seeking medical attention during the pandemic because of COVID-19 fear, especially individuals in vulnerable groups; halting of national screening programs; suspension of cancer follow-up programs, and conversion of many healthcare encounters to telehealth [6]. In the UK, for example, urgent referrals for suspected cancer that would normally follow the 2-week wait target have significantly reduced (>50%), increasing the concerns about a rise in delayed diagnoses. A delay in diagnosis may also result in presentation of increased numbers of patients with more advanced stage disease. This has significant implications in service planning, as these patients are likely to require primary systemic therapy as the initial treatment option or might need more extensive and complex surgery with impact on theater time and capacity.

The use of technology and new patient pathways would help manage the expected increase in service demand. Telehealth is more widely accepted by patients, with the majority being open to a non-face-to-face doctor’s visit due to the convenience of more accessible care. Clinicians have found telehealth to be associated with increased patient engagement and satisfaction, improved quality of care and a reduction in appointment no-shows, transportation expenses and overrunning clinics. However, telehealth consultations are associated with an increased cost for outlay and training of the technology and variable levels of trust and willingness to adopt and use the technology. Clinicians also report that there is a difficulty in building the clinician–patient relationship due to the absence of a ‘smile,’ ‘physical examination’ or ‘nonverbal communication’ cues that are inherently important and are nonquantifiable human factors.

Moving into the recovery phase, although there is an expected increase in feasibility of face-to-face consultations, telehealth can still be integrated into the patient pathway; outpatient referrals can be triaged initially and followed by telephone assessment or video consultations or a direct-to-test approach. If required, a face-to-face consultation can be organized thereafter and reduces the risk of an underappreciation of symptoms [6]. For breast cancer patients, the initial consultations for ‘breaking bad news’ requires a face-to-face encounter. However, for routine follow-up appointments where the physical examination is not as urgent, then video consultations are an acceptable alternative. In addition, the issue of privacy cannot be underestimated and standards for practice should be equitable for all consultations, whether in person or virtual. Face-to-face consultations will require suitably sized clinic rooms to enable social distancing for the patient and clinician, availability of appropriate personal protective equipment, regular cleaning to follow national and local infection control guidelines and as access to systems for patients with additional needs such as disabilities and language interpreters.

The prioritization of surgical waiting lists will need to be done on a department and individual clinician basis following national guidelines [14–18]. The close collaboration between specialties and the role of the multidisciplinary meetings are crucial in leading the discussions for prioritization of patients on waiting lists. Use of scoring systems such as MeNTS could be a helpful tool in evaluating the required hospital resources, the effect of treatment delay on a patient’s underlying disease and the risk the procedure poses for the surgical team [19].

Minimizing the risk of COVID-19 while continuing optimal breast cancer care during the recovery phase can be mitigated to some extent by centralization of healthcare services and use of specialized clinical networks or cancer hubs. The aim of these hubs is to offer administrative and organizational infrastructure so that hospitals across a region can work together to maximize capacity by matching patients to the right expert clinical team or bringing the patient to the treatment site while remaining under the care of the patient’s own specialist team. This approach has several advantages: the hubs pool modest volumes of protected resources while concentrating a high volume of cases, are usually ‘COVID-19-protected’ due to preoperative staff and patient screening, and are being kept separate from units providing essential critical care for the acutely unwell, thus lowering the risk of contracting the virus. The paradigm of a breast cancer hub was successfully employed in Milan, Italy, one of the epicenters of COVID-19 until recently, where more than 350 operations were performed in 1 month, a 20% increase compared with previous activity, with no reported cases of SARS-CoV-2 infection [20]. A similar approach for cancer care has also been employed in London, UK, where services are now coordinated by a specialist ‘cancer hub’ to ensure National Health Service hospitals continue to deliver as much cancer treatment as possible and people receive the treatment they need [21].

In light of the recommendations and warnings outlined by Bill Gates [22], there will be permanent social, cultural and behavioral changes after this global catastrophe. This will influence the evolution of a risk-stratified approach to breast cancer care with an aim to reduce the risk of COVID-19 to patients and healthcare workers, assessing the competing risks of complications and death associated with COVID-19 infection while balancing the risk of disease progression and treatments of marginal benefits, and evaluating modifications to current treatments.

The ability to escalate and de-escalate treatment pathways will be needed to deal with the inevitable waiting lists and the predicted surge in cancer cases, and as such, future capacity planning is crucial within the current commissioning arrangements. Multidisciplinary cancer services will need to work and act cooperatively to quantify backlogs, forecast demand and load balance across tumor biology.

Only by applying an evaluated and robust risk stratifying approach to cancer patients and working collaboratively will there be a unified approach to cancer care immediately following and for the anticipated peaks of COVID-19 so that breast cancer surgery will not stop again. The urgent reorganization and allocation of healthcare resources, staff and infrastructure that has occurred over the past 3 months has been remarkable and could be the foundation of novel future working practice. Cancer hubs might play a pivotal role by providing centralized breast cancer care to ensure capacity and demand is matched, particularly in meeting national cancer targets such as the 31 diagnosis and 62 treatment day targets in the UK, or simply by acting as coordinators to provide oversight and governance and ensure transparency and equity of care.
